# Abutment on Titanium-Base Hybrid Implant: A Literature Review

**DOI:** 10.1055/s-0042-1750801

**Published:** 2022-10-11

**Authors:** André Luiz de Melo Moreno, Daniela Micheline dos Santos, André Pinheiro de Magalhães Bertoz, Marcelo Coelho Goiato

**Affiliations:** 1 Department of Dental Materials and Prosthodontics, São Paulo State University, School of Dentistry, Araçatuba, São Paulo, Brazil

**Keywords:** implants, implanted, Ti-base pillar

## Abstract

An increase in the use of Computer-Aided Design (CAD) and Computer-Aided Manufacturing (CAM) technologies challenges the conventional prosthetic fabrication procedures that are practical and centered on a digital workflow for the patient, especially for dental implants. Increasing workflow of digital restoration work, considering computer-used CAM for restoration technology systems and also fast/CAM for building restoration technology; fast/CAD, also known as abut-Base, has increased interest. Studies on adaptation of different restorative materials, on titanium (Ti)-base abutments, traction, and transformed cycling have become relevant. The objective of this work was to research, through literature studies, on restoration-type abutments. A total of 24 articles were found after searching the following terms in PubMed/Medline, Scopus, and Embase databases: “ti-base AND abutment.” Twenty-one manuscripts selected from the inclusion and exclusion criteria. After an analysis of these articles, it was concluded that the Ti-base abutment and components from the same manufacturer as the Implant should be used preferably; milled monolithic crowns designed to adapt to the Ti-base the hybrid abutment-crown assembly does not affect torque maintenance after thermal aging; the saliva and cleaning protocol of the Ti-base bonding surfaces can influence the operations of the Ti-base crowns; Ti-base and Crown surface treatment is recommended for better applicability and stability results, and the superiority of resin-based cements compared with other types of cements.

## Introduction

The use of intraoral scanning and the Computer-Aided Design (CAD) and Computer-Aided Manufacturing (CAM) technologies has increased over conventional prosthetic fabrication procedures, due to the practicality and centered on a fully digital workflow for the patient, especially in implant dentistry. 1 Loosening of the abutment or loosening of the occlusal screw is the most common technical complication, followed by loss of crown retention and screw and abutment fracture. Consequently, current research in implant dentistry is focused on improving prosthetic longevity and ease. Considering the increasing use of digital workflow in restorative dentistry, the use of abutments with computer-aided geometry stored by CAD/CAM technology systems for rapid restoration fabrication, also known as titanium (Ti)-base abutments, has gained interest. The reason behind its indication lies in the possibility of conventional or digital transfer of the implant for the design of monolithic or two-layer crowns of any material that will be extraorally cemented and then screwed to the implant. 2 Typically, two-piece implants can be screwed or cemented to standard or custom abutments. The advantages of using cement-retained restorations are primarily esthetics, passive crown adjustment, reduced sensitivity of the laboratory technique, and the potential for improved load distribution during function. However, a major disadvantage of cement retention is the difficulty in removing excess cement from the gingival sulcus which can lead to peri-implantitis. 3 Cemented prostheses are also indicated to correct improper implant positioning and to improve occlusion control, for example, in narrow implants. On the other hand, the advantages of screw-retained restorations include reversibility which avoids the need for complex procedures during prosthesis removal for maintenance, oral hygiene assessment and peri-implant probing, repairs, or abutment screw tightening. 3 Ti-base are prefabricated abutments with a hybrid concept of cemented and screwed fixation in the same prosthesis where the implant-abutment connection is used with the precision provided by the manufacturer. 3 Implant abutments that are adapted for CAD/CAM use, such as the Ti-base, allow the digital design and milling of customized restorations to be extraorally cemented and screw-retained to the implant. 1 4 Furthermore, currently the most common CAD/CAM systems have a growing database library for rapid fabrication of prostheses on Ti-base 3 abutments. The advantages of this technique include customization of the emergence profile, time efficiency with cost reduction, hybrid retention mechanism (cemented and screwed) that allows removal of excess cement, and improved light curing of the restoration margins before screwing. 1 5 The objective of this study was to carry out a literature review on the topic, hybrid abutment on Ti-base implant.

## Methods

The present work consisted of a literature review where the search for manuscripts was performed in the PubMed/Medline, Scopus, and Embase databases, for articles performed until December 2021. The search strategy used was “ti-base AND abutment”. The inclusion criterion was studies that evaluated the Ti-base abutment in several aspects due to the low number of articles on the topic, published in English. As exclusion criteria, the authors used articles in which they used only Ti abutments (customized or cast), and did not involve the Ti-base-type prefabricated Ti abutment in their study. Manuscripts of the *in vitro* study were selected following evidence-based laboratory medicine. These principles are as follows: (1) asking the question, (2) searching for evidence, (3) appraising the evidence, (4) applying the evidence, and (5) assessing the experience, 6 with the exception of the clinical manuscript by Rathe et al. 7

## Results

**Fig. 1:**
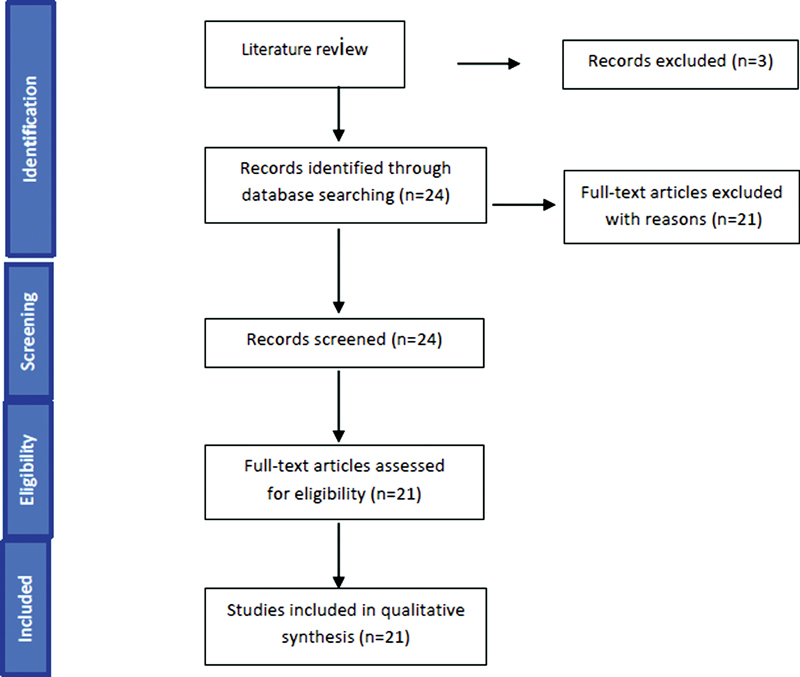
Search strategy of this study.

During the search in the database, 24 manuscripts were found in total, and 21 of these articles were selected from reading the title and abstract, because in the studies, they used the prefabricated abutment of the Ti-base type. The 21 manuscripts were included in this review. The search strategy is detailed in Fig. 1 . The individual details of the studies included in this review can be seen in Table 1 .

**Table 1:** Misfits between the Ti-base abutment/implant and Ti-base abutment/crown

Autor	Objectives	Conclusion
Cardoso et al (2020) 1	Assessed the misfit between Ti-base abutments and implants using the polyvinylsiloxane replica technique using microcomputed tomography	Some Ti-base abutments manufactured by companies other than the implant manufacturer may be more unfit
Camós-Tena et al (2019) 8	They compared the most appropriate restoration technique to obtain the lowest misfit value between the studied prostheses	The milled group statistically showed the best marginal fit. On the other hand, the fully sintered group had the worst results; overcast abutments and Ti-base also presented good results above the casted ones and all systems had gaps below 150 μm (specifically below 60 μm), so clinically they are all good options for rehabilitation
Ramalho et al (2020) 9	Evaluated the effect of different workflows for abutment fabrication on the internal fit at the implant-abutment interface	Prefabricated Ti-base and UCLA abutments exhibited better internal fit at the implant/abutment connection compared with abutments fabricated through a fully digitized workflow (custom CAD/CAM abutments)

Abbreviations: CAD, Computer-Aided Design; CAM, Computer-Aided Manufacturing; Ti, titanium.

The selected studies are dated from 2013 to 2021. A generic analysis of the various topics analyzed in the manuscripts was performed being subdivided as follows: (1) misfits between the Ti-base abutment/implant and Ti-base abutment/crown ( Table 1 ); (2) loosening of the abutment or prosthesis screw ( Table 2 ); (3) crown height x screw stability effect ( Table 3 ); (4) Ti-base abutment height effect x cement type ( Table 4 ); (5) tensile and fatigue test ( Table 5 ); and (6) inflammatory effects of individualized abutments cemented in Ti-base ( Table 6 ).

**Table 2:** Loosening of the abutment or prosthesis screw

Autor	Objectives	Conclusion
Al-Zordk et al (2020) 10	Investigated the effect of thermal aging on torque maintenance in crowns with hybrid zirconia, lithium disilicate and PEEK abutments	Hybrid abutment-crown material (zirconia, lithium disilicate, and PEEK) does not affect torque maintenance after thermal aging

Abbreviation: PEEK, polyether ether ketone.

**Table 3:** Crown height effect x screw stability

Autor	Objectives	Conclusion
Yilmaz et al (2021) 11	Evaluated the effect of the height of zirconia implant crowns screwed onto a titanium base (Ti-base) on screw stability after cyclic loading	In internal connection implants, crown height did not affect detorque values, and 14-mm crowns had similar torque loss to shorter crowns after cyclic loading. The survival time to failure of the 14-mm crown-implant complex was shorter, also resulting in fractures of screws and implants.

**Table 4:** Effect height of titanium (Ti)-base abutment x cement type

Autor	Objectives	Conclusion
Silva et al (2018) 2	To evaluate the effect of different Ti-base abutment heights and cement type on traction retention in zirconia-based restorations	They concluded that although the heights of Ti-base abutments did not influence the retentivity of zirconia superstructures, resin-based cements showed significantly greater retention than glass-ionomer and temporary cements
Zahoui et al (2020) 12	Aimed to evaluate the effect of Ti-base height, type of resin cement and surface pretreatment in Y-TZP and/or Ti-base abutment on the traction retention of implant-supported crowns in Y-TZP	Ti-base abutment height influenced the tensile strength of implant-supported CAD/CAM zirconia crowns, where tall abutments had greater retention strength than short ones

**Table 5:** Tensile and fatigue test

Autor	Objectives	Conclusion
Spitznagel et al (2021) 13	This *in vitro* study investigated the effect of retention mode on Ti-base abutments (screw-retained vs. cement-retained) and application of fatigue on the failure load of single-unit lithium disilicate monolithic implant-supported crowns	Pressed or milled monolithic LDS crowns cemented in the Ti-Base abutment or LDS crowns cemented in custom ceramic abutments withstood physiological chewing forces after artificial aging in a simulated mouth of 5 years and had an equally high probability of survival
Nossair et al (2014) 14	Evaluated the fatigue and fracture strength of CAD/CAM zirconia crowns cemented to custom zirconia abutments + Ti-base versus zirconia crowns applied to custom zirconia abutments + Ti-base	CAD/CAM zirconia crowns cemented to custom implant zirconia abutments demonstrated greater fracture resistance compared with applied ceramic crowns
Lopes et al (2018) 3	They evaluated the pullout strength of milled temporary resin crowns, zirconia, titanium and Co-Cr crown materials cemented to the base of Ti-base abutments	The evaluation of data according to the type of cement demonstrated the superiority of resin-based cements in relation to the provisional and baseline groups. Co-Cr and titanium crowns showed higher levels of retention to the Ti-base abutment after being cemented
Zahoui et al (2020) 12	This study sought to evaluate the effect of Ti-base height, resin cement type and surface pretreatment in Y-TZP and/or Ti-base abutment on the traction retention of implant-supported Y-TZP crowns	They concluded that conventional resin cements associated with self-etching adhesive exhibited greater retention than self-adhesive cements; and that surface blasting of Ti-base and tribochemical zirconia silica coating (SB + TBS) increased retention of zirconia crowns, followed by surface blasting of Ti-base (SB) or tribochemical silica coating (TBS)
Salginci et al (2021) 15	Investigated the tensile strength of zirconia, PEEK (polyether ether ketone) and PVDF (polyvinylidene difluoride) materials for hybrid crowns with Ti-Base abutment and monolithic zirconia crown	The tensile strength of Ti-base PVDF abutments was higher compared with zirconia and PEEK abutments
Elshiyab et al (2018) 16	They investigated the fatigue strength and post-fatigue fracture loading of all-ceramic crowns (monolithic lithium disilicate and monolithic zirconia) in hybrid abutment systems in the molar region	Monolithic zirconia crowns had significantly higher fracture strength compared with monolithic crowns made of lithium disilicate. Monolithic zirconia crowns cemented in hybrid abutments should have satisfactory clinical performance, supporting molar masticatory forces
Cardenas et al (2020) 17	Assessed the fatigue strength and failure mode of anterior single-unit restorations using different types of esthetic abutments	TiZirLd abutments have much higher fatigue strength than ZirLd and can be recommended as an esthetic alternative to restore single implants in the anterior region
Gehrke et al (2013) 18	Assessed the retention of CAD/CAM zirconia restorations to Ti-base abutments after artificial aging under simulated oral conditions	The use of resin-based luting agents in combination with air abrasion of the bonding surfaces of Ti-base abutment inserts and CAD/CAM zirconia copings led to sufficient and stable retention of the two parts
Kemarly et al (2019) 19	They evaluated various surface treatments in different combinations and their effects on the tensile bond strength of lithium disilicate to Ti-base	Mechanical roughening with Al _2_ O _3_ air abrasion or CoJet silicoating for lithium disilicate and Ti-base is recommended. Once mechanically modified, Monobond Plus appears to be the superior chemical primer of materials tested for treating Ti-base when using MultiLink hybrid abutment cement
Pitta et al (2020) 20	They evaluated the influence of airborne particle abrasion methods on the surface of Ti-base on bond stability and adhesive retention forces in monolithic lithium disilicate cemented crowns after artificial thermomechanical aging	251/5,000 Resultados de tradução Mechanical treatment of the titanium surface increased the bonding interface stability and retention forces between the Ti-base abutments and the crown and the use of APA with 50-mm Al _2_ O _3_ provided the most stable bonding interface among the methods tested
Burkhardt et al (2021) 5	They evaluated the influence of saliva contamination on Ti-base bonding surfaces on the retention forces of lithium disilicate crowns and investigated the effect of different cleaning protocols	It showed that the retention forces of lithium disilicate crowns on Ti-base abutments were significantly influenced by saliva contamination and titanium surface cleaning; No differences were found between the different types of cleaning protocols that were applied after contamination

Abbreviations: CAD, Computer-Aided Design; CAM, Computer-Aided Manufacturing; Co, cobalt; Cr, chromium; Ti, titanium; TiZirLd, Ti-Bbase with custom zirconiaZr abutment and lithium disilicate crown;

**Table 6:** Inflammatory effects of individualized abutments cemented in titanium (Ti)-base

Autor	Objectives	Conclusion
Rahte et al (2021) 7	Studied the inflammatory effects of individualized Ti-base cemented abutments, evaluated radiographically and clinically	In selected patients (selected to minimize the development of peri-implant disease) with adequate plaque control, the effects on the inflammatory state of the peri-implant tissues do not differ between individualized Ti-base bonded abutments compared with individualized one-piece abutments

## Discussion

An important factor that affects the biomechanical behavior of implant-supported reconstructions is the implant-abutment fit, as the forces on such surfaces are maximized and more stable the smaller the gaps are present. Cardoso et al 1 evaluated the misfit between Ti-base abutments and implants using the polyvinylsiloxane replica technique, using microcomputed tomography (μCT). The results of their study showed a significantly smaller volume gap for the Ti-base abutment of the Control S.I.N Implants (0.67 ± 0.29 mm ^3^ ) and Singular Implants (0.69 ± 0.28 mm ^3^ ) group compared with Odontofix LTDA (1 0.42 ± 0.28 mm ^3^ ) and EFF Dental Components groups (1.04 ± 0.28 mm ^3^ ; *p* < 0.033), with no significant difference between them ( *p* = 0.936). While gap values were homogeneous in the central region, the EFF group showed a significantly higher value in the marginal gap. Consequently, the control group SIN and singular Ti-base abutments showed better volumetric and marginal fit compared with Odontofix and EFF. The study concluded that the Ti-base abutments of the SIN and singular control groups showed better volumetric and marginal fit compared with the Odontofix and EFF groups. Therefore, some Ti-base abutments manufactured by companies other than the implant manufacturer may be more unfit. The abutment fabrication method influenced the implant-abutment interface misfit.

CAD/CAM fabricated zirconia (Zr) abutments, like all ceramic parts, will have lifetimes and performance determined by design details and processing defects. The authors in their study sought to determine whether there were differences in performance attributable to design or fabrication and, if so, how those differences translate into a life expectancy for the restoration. Based on their study, neither all-Zr nor Ti-base abutments were superior. 21 They also concluded that the manufacturer is important; for both Zr- and Ti-base abutments, parts from different manufacturers, design, and manufacturing differences influenced performance and appeared extremely similar on clinical examination. Finally, design defects/problems were suggested for all systems.

The presence of misfits in implant-retained restorations can generate high levels of stress at the implant-bone interface that can compromise osseointegration. Of the implant and generate mechanical and biological complications. By the hybrid crown rehabilitation technique, each compatible Ti-base is designed by a specific implant system which means that each implant brand has its own Ti-base that guarantees the perfect fit. Camós-Tena et al 8 compared the most appropriate restoration technique to obtain the lowest misfit value between the studied prostheses. From the descriptive results, it was observed that the Milled technique presented the best fit in almost all points; on the other hand, polyether ether ketone (PEEK) and sintered presented the lowest. All techniques have good marginal fit ranges: there was no group at maximum results that exceeded 150 μm. The authors therefore concluded that the reamed group statistically presented the best marginal fit. On the other hand, the sintered group had the worst results; overcast abutments and Ti-base also presented good results above the casted ones and all systems had gaps below 150 μm (specifically below 60 μm), so clinically they are all good options for rehabilitation.

Ramalho et al 9 evaluated the effect of different workflows for abutment fabrication on the internal fit at the implant-abutment interface and to correlate two-dimensional (2D) and three-dimensional (3D) misfit measurements. The results of their study showed that the method of abutment fabrication influenced the internal fit at the implant-abutment interface and the fully digital workflow resulted in a less-favorable internal fit compared with the casting method (UCLA) UCLA-type abutments and Ti-base abutments. 3D measurement for internal discrepancy quantification was strongly correlated with 2D measurements. As a result, they were able to conclude that Ti-base and UCLA prefabricated abutments exhibited better internal fit in the implant/abutment connection compared with fully digitized abutments (custom CAD/CAM abutments).

One of the most critical mechanical complications is loosening of the abutment or prosthesis screw. Currently, the incidence of screw loosening ranges from 7 to 11%. The loosening of the screw can cause an imbalance in the distribution of occlusal forces, fracture of the screw and the implant, microspaces between the abutment and the implant that can allow the entry of bacteria that will affect osseointegration. 10 Al-Zordk et al 10 investigated the effect of thermal aging on torque maintenance in crowns with hybrid Zr, lithium disilicate and PEEK abutments. In addition, the fracture strength of Zr, lithium disilicate, and PEEK in hybrid abutment crowns. The authors concluded the following: (1) the hybrid abutment-crown material (Zr, lithium disilicate, and PEEK) does not affect torque maintenance after thermal aging; (2) based on the fracture load, zirconia hybrid-abutment-crown can be used, while lithium disilicate and PEEK hybrid abutment-crown may cautiously serve in the premolar region.

Yilmaz et al 11 evaluated the effect of the height of screw-retained Zr implant crowns on a Ti-base on screw stability after cyclic loading and to investigate the survival of screw-retained Zr crowns on Ti-base implants of different heights after cyclic loading. The authors concluded that (1) in internally connecting implants, crown height did not affect detorque values and 14-mm crowns had similar torque loss to shorter crowns after cyclic loading; and (2) the survival time to failure of the 14-mm crown-implant complex was shorter, also resulting in fractures of screws and implants.

Silva et al 2 evaluated the effect of different heights of Ti-base abutments and type of cement on traction retention in Zr-based restorations and had as results that the Ti-base abutment of 4-mm height (tall) demonstrated retention similar to 2.5-mm high (short) abutments when data are collapsed on cement type ( *p* > 0.74). On the other hand, resin-based cements (U200 and Ultimate) showed significantly higher tensile values compared with temporary and glass ionomer cements ( *p* < 0.01), with no significant difference between U200 and Ultimate and between groups. Provisional and glass ionomer ( *p* > 0.34). Therefore, they concluded that although the heights of Ti-base abutments did not influence the retentivity of Zr superstructures, resin-based cements showed significantly greater retention than glass-ionomer and temporary cements.

Spitznagel et al 13 in their *in vitro* study investigated the effect of retention mode on Ti-base abutments (screw-retained vs. cemented) and application of fatigue on the load failure of implant-supported monolithic single lithium disilicate crowns. Pressed or milled, screw-retained monolithic monolithic lithium disilicate (LDS) crowns cemented directly into Ti-base abutments or LDS crowns cemented into custom ceramic abutments withstood physiological chewing forces after artificial aging in a simulated mouth for 5 years and had an equally high probability of survival. However, a significant decrease in the load to the Failure was observed in LDS crowns cemented on custom ceramic abutments after fatigue.

Nossair et al 14 evaluated the fatigue and fracture resistance of CAD/CAM Zr crowns cemented to custom Zr abutments + Ti-base versus Zr crowns applied to custom Zr abutments + Ti-base. The authors concluded that within the limitations of the study, CAD/CAM Zr crowns cemented on custom implant Zr abutments demonstrated greater fracture resistance compared with hand-cast and bonded crowns.

Milling strategies between different denture materials vary and can produce discrepancies in the cementation space which eventually influence the final retention of the crown. Lopes et al 3 sought to evaluate the pullout strength of milled temporary resin crowns, Zr, Ti, and cobalt/chromium (Co-Cr) crown materials cemented to the base of Ti-base abutments. The evaluation of their study data as a function of cement type demonstrated the superiority of resin-based cements over the provisional and baseline groups ( *p* < 0.01). While Co-Cr crown had the highest pullout strength values and provisional resin (Pr) crowns had the lowest values (data collected on the cement; *p* < 0.001). Retention data as a function of both factors showed similar pullout strength between the cementless groups ( *p* < 0.001), except for the baseline Zr group. In addition, Co-Cr showed higher pullout strength compared with other materials. The study concluded that both crown material and cement type influenced the pullout strength of implant-supported CAD/CAM crowns on Ti-base abutments. Co-Cr, and Ti crowns showed higher levels of retention to the Ti-base abutment after being cemented. The self-adhesive resin cement exhibited superior behavior when compared with the temporary cement and no cement groups, regardless of crown material.

Zahoui et al 12 sought to evaluate the effect of Ti-base height, type of resin cement, and Yttria-stabilized tetragonal zircônia polycrystal (Y-TZP) (Zr) surface pretreatment and/or Ti-base abutment on the traction retention of implant-supported **Y** -shaped crowns, TZP. The authors concluded in their study that the height of the Ti-base abutment influenced the tensile strength of implant-supported CAD/CAM Zr crowns, where tall abutments had greater retention strength than short ones. They also concluded that the conventional resin cements associated with self-etching adhesive exhibited greater retention than self-adhesive cements. Finally, they concluded that surface blasting of Ti-base and tribochemical Zr silica coating (surface blasting with alumina particles [SB] + tribochemical surface blasting [TBS]) increased the retention of Zr crowns, followed by surface blasting of Ti-base (SB) or tribochemical coating of Zr. Silica (TBS) compared with the no treatment (NT) surface treatment group. Hierarchically, the results demonstrated a direct relationship between Ti-base height, micromechanical, and/or chemical pretreatment (SB + TBS) and adhesive cementation with conventional resin cement associated with self-etching adhesive to maximize retention of Zr crowns.

Salgıncı et al 15 investigated the tensile strength of Zr, PEEK, and polyvinylidene difluoride (PVDF) materials for hybrid crowns with Ti-base abutment and monolithic Zr crown when used in implant-supported restorations. The authors concluded that (1) the tensile strength of PVDF Ti-base abutments was higher compared with Zr and PEEK abutments; (2) although there is an indication of MultiLink cement for hybrid abutment, in this study, cementation of two-piece abutments with Ti-base was done and the strength of this cement to PEEK abutments was insufficient.

Elshiyab et al 16 investigated the fatigue strength and postfatigue fracture loading of all-ceramic crowns (monolithic lithium disilicate and monolithic Zr) in hybrid abutment systems, in the molar region. Results of this study showed that monolithic implant-supported crowns made of Zr had significantly higher fracture strength compared with monolithic crowns made of lithium disilicate. The results also suggested that the application of fatigue caused a significant reduction in the fracture strength of both all-ceramic groups. The study concluded that monolithic implant-supported Zr restorations cemented in hybrid abutments are unlikely to fracture and should have satisfactory clinical performance, supporting molar masticatory forces.

Tribst et al 22 evaluated the influence of the modulus of elasticity of materials (Zr, lithium disilicate [D], and hybrid ceramic [H]) on the stress distribution of cemented prostheses in hybrid implant-supported abutments (cone Morse). In their study, it was possible to observe that a greater concentration of stress in the cervical region occurred inversely proportional to the elastic modulus of the crown, and directly to the elastic modulus of the Ti-base hybrid abutment. The peak tensile stress occurred in the cervical area of the crown notch surface. With the von-Mises tension criteria analyzing the hybrid abutment, all groups had a probability of failure in the cervical region of the metallic connection. Statistical analysis of stress peaks in the crown and abutment analysis of variance (ANOVA) showed a significant difference in the stress distribution between the evaluated combinations. Low-stress concentration occurred with Zr crowns, and hybrid abutment for crowns with lower elastic modulus. The authors, considering this theoretical study for Morse taper implants, concluded that the association of a rigid crown with a resilient hybrid abutment reduces the concentration of tensile stress in the cervical region of the restoration.

Cárdenas et al 17 evaluated the fatigue strength and failure mode of implant-supported anterior single restorations using different types of esthetic abutments and concluded the following: (1) the fatigue strength and failure mode of different abutments are influenced by the type of restorative material and abutment design. Ti-base with custom Zr abutment and lithium disilicate crown (TiZirLd) withstood the highest values of fatigue strength, although its failures were catastrophic. The Ti-base with polymer-infiltrated ceramic abutment and crown (TiEn) group had the lowest fracture strength values, however, failure did not affect the Ti-base or screw; (2) all groups have the potential to resist the physiological occlusal forces that occur in the anterior region; however, TiEn is not recommended, as all specimens were fractured with a small number of cycles, suggesting limited survival; (3) TiZirLd abutments have much higher fatigue strength than custom Zr abutments with lithium disilicate crown (ZirLd), and therefore can be recommended as an esthetic alternative to restore single implants in the anterior region; and (4) careful attention should be paid when choosing TiZirLd for patients at high risk of fracture, since the failure mode of this group can be catastrophic, making it difficult to back off.

According to Gehrke et al, 18 surface conditioning methods and the size of the cement gap can have a significant influence on the retention of a Zr abutment/coping bonded to a secondary Ti abutment and the retention strength can be influenced. by the type of cementing material. In their study, they evaluated the retention of CAD/CAM Zr restorations to Ti-base abutments after artificial aging under simulated oral conditions using three different types of resin-based luting agents. The Kruskal–Wallis test does not indicated a significant difference between the retention values of the cementing agents tested ( *p* = 0.1314). The failure modes of all two-piece Ti-base/coping Zr abutments tested were completely adhesive, leaving the detached Zr coping and the Ti abutment intact. Based on the results of this study, the use of resin-based luting agents in combination with air abrasion of the bonding surfaces of Ti-base abutment inserts and CAD/CAM Zr copings led to sufficient and stable retention of the two parts. The bond stability of the investigated luting agents exceeded the general fracture strength limits of two-piece Zr coping and Ti-base abutments. A notable difference between the mean retention values of the tested luting materials was demonstrated. However, statistical analysis revealed that this difference was not significant. The silica nature of lithium disilicate allows for surface treatment with hydrofluoric acid prior to silanization. The polycrystalline nature of Zr may require other surface treatment methods that may rely more on mechanical rather than chemical retention. The authors evaluated several surface treatments in different combinations and their effects on the tensile bond strength of lithium disilicate to the Ti-base implant and concluded that, due to the limitations of this study, regarding the binding of lithium disilicate copings to Ti-base, mechanical roughening with Al _2_ O _3_ air abrasion or CoJet silicoating is recommended. Once mechanically modified, Monobond Plus appears to be the superior chemical primer of materials tested for treating Ti-base when using MultiLink hybrid abutment cement. The success of the Ti-base abutment concept may be dependent on the stability of the bonding interface between the Ti-base and the ceramic components. To achieve high and durable adhesive retention, different surface pretreatments have been proposed. Despite a well-established bonding protocol for glass ceramics, the best mechanical surface treatment to apply to Ti-base is still unclear (Pitta et al). 20 The authors evaluated the influence of airborne particle abrasion methods on the surface of Ti-base on bond stability and adhesive retention forces in monolithic lithium disilicate cemented crowns after artificial thermomechanical aging. In the results of their study, they were able to verify that the stability of the bonding interface was influenced by the method of abrasion with applied air particles ( *χ* 2 = 13.889, df = 3, *p* < 0.05). The application of a airborne-particle abrasion (APA) method had a significant effect on the holding force (N) values ( *p* < 0.05). The authors therefore concluded that mechanical treatment of the Ti surface increased the bonding interface stability and retention forces between the Ti-base abutments and the crown and the use of APA with 50-mm Al _2_ O _3_ (aluminum oxide) provided the most stable union interface among the tested methods.

Studies have shown that the adhesive cementation of the restoration to a Ti-base abutment can be predictably achieved by applying an air particle abrasive treatment to the surface of the Ti-base abutment and etching the surface of the Ti and ceramic abutment with a respective primer followed by resin-based cement. Primers are used to promote adhesion between different substrates. Although primers are substrate-specific, universal silane-based primers can be used with both ceramic and metal substrates as long as the bonding mechanism is similar. In general, a satisfactory union is obtained in a controlled and extraorally clean situation. However, in some clinical situations, contact with oral fluids, such as saliva, can hardly be avoided, such as during digital impression at the abutment level or during the testing procedure and restoration adjustments before cementation. 4 Burkhardt et al 4 evaluated the influence of saliva contamination on the Ti-base abutment bonding surfaces on the retention forces of lithium disilicate crowns and investigated the effect of different cleaning protocols and showed that retention forces of lithium disilicate crowns over Ti-base abutments were significantly influenced by saliva contamination and Ti surface cleaning; however, no difference was found between the different types of cleaning protocols that were applied after contamination.

Due to the intimate contact between the abutment and the implant, the physical and chemical characteristics of the bonding material can affect peri-implant inflammatory reactions. In line with this, the objective of using an individualized Ti abutment instead of Zr was to avoid a second material which could influence the peri-implant tissue reaction. Rahte et al 8 studied the inflammatory effects of individualized Ti-base cemented abutments, evaluated radiographically, clinically, and, additionally, by biomarkers. Within the limits of the study, they were able to conclude that in selected patients (selected to minimize the development of peri-implant diseases) with adequate plaque control, the effects on the inflammatory state of the peri-implant tissues do not differ between individualized abutments bonded in Ti-base in compared with individualized one-piece abutments.

## Conclusion

The Ti-base abutment and components from the same manufacturer as the implant must be used; salivary administration and cleaning of adhesive surfaces can function as the protocol for adhesive surfaces. The Ti-base hybrid implants are a good indication for rehabilitations.
